# Supplementary material for: Integrated OMICs Approach for the Group 1 Protease Mite-Allergen of House Dust Mite Dermatophagoides microceras
Source: Int J Mol Sci. 2022 Mar 30;23(7):3810. doi: 10.3390/ijms23073810 (PMC8998267; doi:10.3390/ijms23073810)
Supplement: Supplementary file 1 [file ijms-23-03810-s001.zip › ijms-1648365-supplementary.pdf]

Supplementary Table S1. The parameters of ABySS.

N	min	N80	N50	N20	E-size	max	sum
503027	31	149	1052	6344	5016	114864	106e6

Supplementary Table S2. The primers for real-timer PCR

Primer	Sequence (5' to 3')
IL-6-for	TTCGGTCCAGTTGCCTTCTC
IL-6-rev	GAGGTGAGTGGCTGTCTGTG
IL-8-for	CTTGTCATTGCCAGCTGTGT
IL-8-rev	TGACTGTGGAGTTTTGGCTG
GAPDH-for	ACCAGCCCCAGCAAGAGCACAAG
GAPDH-rev	TTCAAGGGGTCTACATGGCAACTG

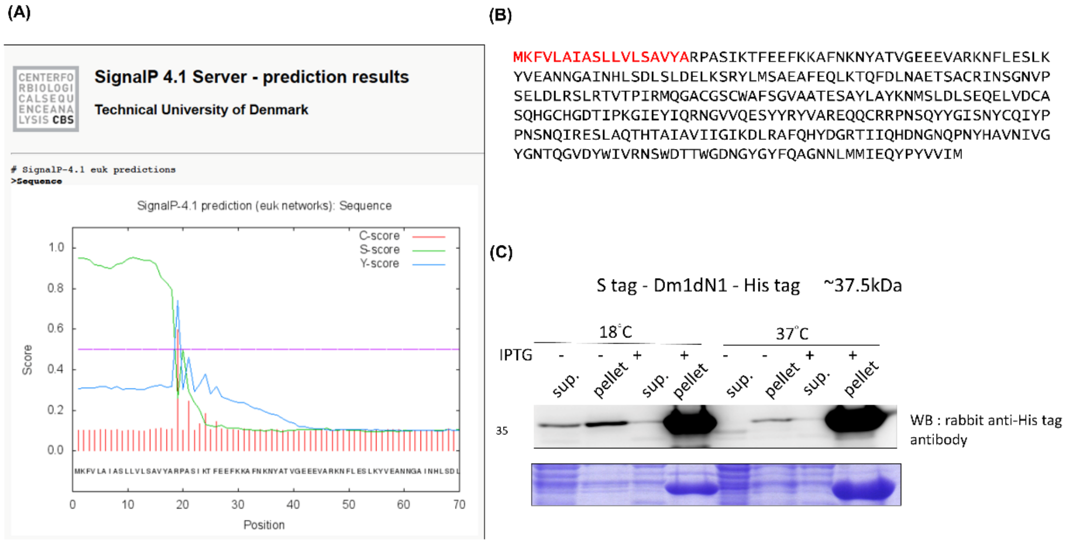

Supplementary Figure S1. The signal peptide prediction and protein production for putative *Der m 1*. (**A**) The protein sequence of *Der m 1* was analyzed by SignalP 4.1 Server, the first 18 residues were predicted as signal peptides as color red. (**B**) and (**C**) The truncated recombinant *Der m 1* (Dm1dN1, 37.5 kDa) was constructed and expressed in the *E. coli* IPTG induction system, and the truncated recombinant *Der m 1* was purified by affinity column for subsequent experiments.
